# PARP14 is a novel target in *STAT6* mutant follicular lymphoma

**DOI:** 10.1038/s41375-022-01641-x

**Published:** 2022-07-18

**Authors:** Michael Mentz, William Keay, Carolin Dorothea Strobl, Martina Antoniolli, Louisa Adolph, Michael Heide, Axel Lechner, Sarah Haebe, Elisa Osterode, Robert Kridel, Christoph Ziegenhain, Lucas Esteban Wange, Johannes Adrian Hildebrand, Tanaya Shree, Elisabeth Silkenstedt, Annette M. Staiger, German Ott, Heike Horn, Monika Szczepanowski, Julia Richter, Ronald Levy, Andreas Rosenwald, Wolfgang Enard, Ursula Zimber-Strobl, Michael von Bergwelt-Baildon, Wolfgang Hiddemann, Wolfram Klapper, Marc Schmidt-Supprian, Martina Rudelius, Deepak Bararia, Verena Passerini, Oliver Weigert

**Affiliations:** 1 Department of Medicine III, Laboratory for Experimental Leukemia and Lymphoma Research (ELLF), Ludwig-Maximilians-University (LMU) Hospital, Munich, Germany; 2 Research Unit Gene Vectors, Helmholtz- Center Munich, German Research Center for Environmental Health, Munich, Germany; 3 Department of Otolaryngology, Ludwig-Maximilians-University (LMU) Hospital, Munich, Germany; 4 Division of Oncology, Department of Medicine, School of Medicine, Stanford, CA USA; 5 Princess Margaret Cancer Centre, University Health Network, Toronto, ON Canada; 6 Anthropology and Human Genomics, Faculty of Biology, Ludwig-Maximilians-University, Munich, Germany; 7 Department of Clinical Pathology, Robert Bosch Hospital, Stuttgart, Germany; 8 Dr. Margarete Fischer-Bosch-Institute of Clinical Pharmacology, Stuttgart and University of Tübingen, Tübingen, Germany; 9 Institute of Pathology, Hematopathology Section, University of Schleswig-Holstein, Kiel, Germany; 10 Institute of Pathology, University of Würzburg and Comprehensive Cancer Centre Mainfranken, Würzburg, Germany; 11 German Cancer Consortium (DKTK), Munich, Germany; 12 German Cancer Research Center (DKFZ), Heidelberg, Germany; 13 Institute of Experimental Hematology, School of Medicine, Center for Translational Cancer Research (TranslaTUM), Technical University of Munich, Munich, Germany; 14 Institute of Pathology, Faculty of Medicine, Ludwig-Maximilians-University, Munich, Germany

**Keywords:** Cancer microenvironment, Cancer genetics

## Abstract

The variable clinical course of follicular lymphoma (FL) is determined by the molecular heterogeneity of tumor cells and complex interactions within the tumor microenvironment (TME). IL-4 producing follicular helper T cells (T_FH_) are critical components of the FL TME. Binding of IL-4 to IL-4R on FL cells activates JAK/STAT signaling. We identified *STAT6* mutations (*STAT6*^MUT^) in 13% of FL (*N* = 33/258), all clustered within the DNA binding domain. Gene expression data and immunohistochemistry showed upregulation of IL-4/STAT6 target genes in *STAT6*^MUT^ FL, including *CCL17*, *CCL22*, and *FCER2* (CD23). Functionally, *STAT6*^MUT^ was gain-of-function by serial replating phenotype in pre-B CFU assays. Expression of STAT6^MUT^ enhanced IL-4 induced *FCER2*/CD23, *CCL17* and *CCL22* expression and was associated with nuclear accumulation of pSTAT6. RNA sequencing identified PARP14 -a transcriptional switch and co-activator of STAT6- among the top differentially upregulated genes in IL-4 stimulated *STAT6*^MUT^ lymphoma cells and in *STAT6*^MUT^ primary FL cells. Quantitative chromatin immunoprecipitation (qChIP) demonstrated binding of STAT6^MUT^ but not STAT6^WT^ to the PARP14 promotor. Reporter assays showed increased IL-4 induced transactivation activity of STAT6^MUT^ at the PARP14 promotor, suggesting a self-reinforcing regulatory circuit. Knock-down of PARP14 or PARP-inhibition abrogated the STAT6^MUT^ gain-of-function phenotype. Thus, our results identify PARP14 as a novel therapeutic target in *STAT6*^MUT^ FL.

## Introduction

Follicular lymphoma (FL) is among the most common malignant lymphomas worldwide. Most patients present with advanced stage disease and are still considered incurable [1]. Although considered the prototype of indolent lymphoma, FL is a highly heterogenous disease and a subset of patients has early treatment failure, aggressive clinical course and remarkably short overall survival [2, 3]. Treatment of relapsed, refractory or transformed FL remains a major clinical challenge [4]. Molecular-targeted therapies hold promise to improve treatment outcome if tailored toward the individual disease biology of distinct patient subsets.

The molecular hallmark of FL is the rearrangement of chromosome 18q21 resulting in overexpression of anti-apoptotic BCL2 [5]. Dozens of additional recurrent gene mutations have been reported in FL, many of which are likely to have distinct implications on the biology and the clinical course of the disease (reviewed in [6]). In addition to tumor cell intrinsic alterations, features of the tumor microenvironment (TME) have also been shown to be associated with treatment outcome [7–11].

FL cells retain a remarkable dependency on the TME, orchestrating a tumor-permissive immune niche (reviewed in [12]). The FL TME is enriched with T follicular helper (T_FH_) cells which express higher levels of IL-4 compared to T_FH_ cells from healthy lymph nodes [13, 14]. Binding of IL-4 to its receptor (IL-4R) on lymphoma cells recruits JAK1/3 and activates STAT6 by phosphorylation of Y641. This promotes the formation of STAT6 homodimers, which translocate to the nucleus and bind to regulatory elements of STAT6 target genes via the DNA-binding domain. In the presence of IL-4, transcriptional repressors are released (e.g., HDAC2 and HDAC3) and transcriptional coactivators are recruited (e.g., EP300, NCOA1 and NCOA2) in a process that involves PARP14, a member of the poly ADP-ribose polymerases (PARP) family, thereby forming the STAT6 enhanceosome that drives STAT6-dependent gene expression [9, 15–17].

Others and we have previously reported that *STAT6* is recurrently and significantly mutated in FL and other B cell lymphomas [18–23]. In-vitro studies and crystal structure analyses have suggested that these mutations might enhance the ability of STAT6 to bind to canonical DNA binding sites of STAT6 [23, 24]. However, the underlying mechanism of how *STAT6* mutations (*STAT6*^MUT^) contribute to FL biology remains incompletely understood. Moreover, potential therapeutic vulnerabilities have not yet been explored. Here we describe a therapeutically targetable PARP14-mediated self-reinforcing regulatory circuit that amplifies IL-4 induced STAT6-dependent gene expression in STAT6^MUT^ lymphoma cells.

## Materials and methods

### Cell lines and reagents

OCI-Ly1and OCI-Ly8 cells were cultured in IMDM (PAN Biotech, Aidenbach, Germany). 293 T HEK cells and HeLa cells were cultured in Dulbecco’s Modified Eagle Medium. All cells were cultured with 10% FBS (PAN), 37 °C 5% CO_2_. Cell lines were authenticated by short tandem repeat analysis (Eurofins, Val Fleuri, Luxembourg) and tested negative for mycoplasma by PCR. Of note, we confirmed that OCI-Ly1 harbors a variant (G375R) in the STAT6 DNA-binding domain [23] that, to the best of our knowledge, has not been reported in any other cell line or primary tumor sample. Cells were stably transduced with a CMV-driven cDNA expression construct (pHAGE-CMV-MCS-IRES-ZsGreen; PlasmID, EvNO00061605) encoding for Flag-tagged mutant (MUT) STAT6 (D419G, D419N, N421K, or D519V) or wild type (WT) STAT6 (PlasmID, HsCD00365550) as previously described [7], and stimulated with human recombinant IL-4 (Miltenyi Biotec, Cologne, Germany) as indicated.

### Human ex vivo FL-like co-culture model

Briefly, we isolated germinal center (GC) cells from human tonsils and immortalized them by transduction of BCL2 and BCL6 as previously described [25]. In addition, we stably expressed STAT6^WT^, STAT6^D419G^ or EV control. These FL-like cells were grown on the follicular dendritic cell (FDC) feeder cell line YK6-CD40lg-IL21, which stably expresses CD40L and IL-21.

### Immunoblotting

Western blot and immunoprecipitation (IP) experiments were performed as previously described [7]. Technical details and antibodies are listed in the supplementary methods. Subcellular fractions were prepared using the Qproteome Nuclear Protein Kit (Qiagen, Hilden, Germany).

### Pre-B CFU assay

Emu-BCL2 (B6.Cg-Tg(BCL2)36Wehi/J) mice were sacrificed and femurs were flushed with phosphate buffered saline (PBS). 1 × 10^6^ bone marrow cells were retrovirally transduced with *STAT6*^WT^, *STAT6*^D419G^ or empty vector (EV) (all cloned into pMSCV-IRES-GFP) as previously [7] described, incubated for 4 h (5% CO_2_, 37 °C) and plated onto methylcellulose (0.3 × 10^6^ cells/mL) that supports the growth of pre-B colony-forming-units (CFUs) (M3630, Stem Cell Technologies, Vancouver, British Columbia, Canada). Pre-B CFUs were counted according to the manufacturer’s guidelines. Cells from each plate were washed off and replated entirely onto fresh M3630 methylcellulose media every 7 days.

### RNA sequencing

OCI-Ly1 cells stably expressing either STAT6^WT^ (*N* = 9) or STAT6^MUT^ (D419G, D419N, N421K, each *N* = 3) were stimulated with IL-4 (10 ng/ml for 20 min), washed and replated in IL-4 free media. RNA sequencing libraries were prepared from mRNA isolated at 2, 4, and 8 h (Direct-zol RNA MiniPrep Plus, Zymo Research, Irvine, California, USA), respectively, using the prime-seq protocol as previously described and sequenced on an Illumina HiSeq 1500 system [26]. Raw sequence data was processed following the Drop-seq data pipeline. Differential gene expression analysis was performed using DESeq2 package [27]. Raw and processed RNA sequencing data have been made available via the Gene-Expression-Omnibus platform under the accession number GSE208031.

We re-analyzed previously published single cell RNA sequencing data [28] from eight primary FL with known STAT6 genotypes using the R package Seurat (version 3) [29].

### Proximity ligation assay (PLA)

Briefly, 2 µm cytoblock sections of OCI-Ly8 cells (STAT6^WT^ vs STAT6^MUT^ with or without IL-4 stimulation [10 ng/ml for 20 min]) were co-stained with mouse anti-STAT6 (LSBio, LS-B6154, 1:800) and rabbit anti-PARP14 (Sigma, HPA012063, 1:100). Images were acquired on a Vectra Polaris imaging system using inForm automated image analysis software (Akoya). PLA spots (TexasRed channel) per cell were counted in five representative view fields (40×) using HALO Image Analysis Platform version 3.2.

### Luciferase reporter assay

*PARP14* was cloned into pGL3 basic vector as detailed in the supplement following the manufacturer’s guidelines (Promega, Madison, Wisconsin, USA). The PARP14-pGL3 vector (200 ng) was co-transfected with pRL-CMV Renilla luciferase control reporter (40 ng, Promega) and 10 or 50 ng STAT6 expression vector (pHAGE CMV-STAT6^MUT^ vs pHAGE-CMV-STAT6^WT^) into 293 T HEK cells using ViaFect^TM^ (Promega). After 24 h, cells were stimulated with IL-4 (10 ng/ml) for 6 h and analyzed by Dual-Glo^®^ Luciferase Assay (Promega). The PARP14 knock-down experiment was performed in HeLa cells since these express high levels of endogenous PARP14. Two shRNA constructs were used to knock-down PARP14 (SHC016-1EA, TRCN0000053158, TRCN0000053159, Sigma Aldrich, St. Louis, Missouri, USA), a non-targeting shRNA construct was used as control (see supplementary methods). PJ34 (Selleckchem, Munich, Germany) was used as a PARP inhibitor (50 µM, 30 min prior to IL-4 stimulation). Viability was measured by Vi-Cell XR, cell viability analyzer (Beckman Coulter).

### Quantitative chromatin immunoprecipitation (qChIP)

qChIP was performed in OCI-Ly8 expressing *STAT6*^WT^, *STAT6*^D419G^ or EV stimulated with IL-4 (10 ng/ml for 24 h). Cells were washed in PBS and double crosslinked with EGS (Sigma Aldrich) and formaldehyde (1%, Thermo Fisher Scientific, Waltham, Massachusetts, USA). 15 µg of sheared chromatin was used for each IP of 3x Flag-tagged STAT6 (STAT6^WT^ or STAT6^D419G^), H3 or IgG as previously described [30].

Additional methods are provided in the supplement.

## Results

### *STAT6* mutations cluster within the DNA binding domain

**Fig. 1:**
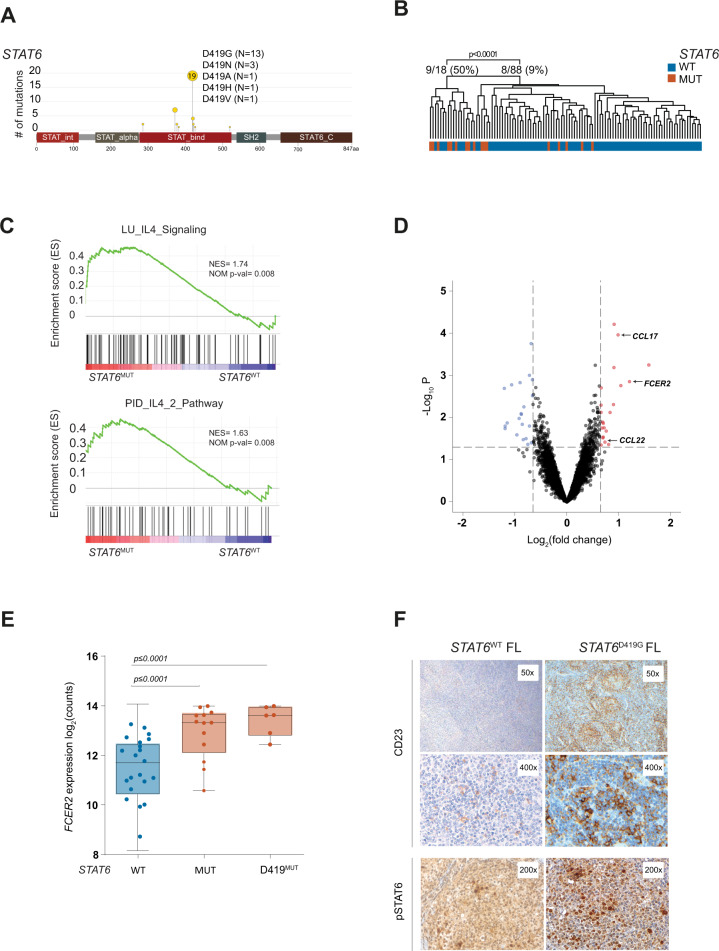
*STAT6* mutations are associated with activated IL-4 signaling in human FL. **A** Lolliplot of *STAT6* mutations in 258 human FL biopsies. **B** Unsupervised hierarchical clustering of gene expression data from 106 human FL, annotated for *STAT6* genotype. **C** Same data used for gene set enrichment analysis (GSEA) for established IL-4 signatures comparing *STAT6*^WT^ *and STAT6*^MUT^. **D** Volcano plot of differentially expressed genes between *STAT6*^WT^ and *STAT6*^MUT^ FL (*N* = 106, log2FC = ±0.65, *p* value = 0.05). **E** *FCER2* (CD23) gene expression in human FL with wild type *STAT6* (*STAT6*^WT^) vs any *STAT6* mutation (*STAT6*^MUT^) vs *STAT6* mutations at position D419 (*STAT6*^D419^). **F** Representative immunohistochemistry (IHC) stains for CD23 and pSTAT6 in human FL with *STAT6*^WT^ (left) and *STAT6*^MUT^ (*STAT6*^D419G^, right).

In a cohort of 258 patients with advanced stage FL, we identified 35 *STAT6* mutations in 33 diagnostic biopsies (13%; Fig. 1A). All mutations clustered within the DNA binding domain, mostly at D419 (N = 16, 43%). In addition, 3 patients harbored a known (yet rare) polymorphic D419N variant at this position (rs11172102, Fig. 1A). The variant allele frequencies (VAF) of two of these cases were suggestive of somatically acquired mutations (Supplementary Fig. 1A). Two FL harbored two mutations each, both located within the DNA binding domain, respectively. Manual review of the sequencing reads spanning the mutations demonstrated that these mutations were located *in cis* on the same allele (Supplementary Fig. 2A).

### *STAT6*^MUT^ FL are enriched for IL-4 linked gene expression

To identify gene expression patterns associated with *STAT6*^MUT^, we re-analyzed the available genome-wide RNA profiling data from 106 diagnostic biopsies from our cohort, comprising 17 and 89 cases with and without *STAT6*^MUT^, respectively (details provided in Supplementary Methods). Unsupervised hierarchical clustering distinguished two groups that were enriched for the respective *STAT6* genotypes (MUT vs WT, *p* < 0.0001; Fig. 1B). Gene set enrichment analysis (GSEA) demonstrated that *STAT6*^MUT^ cases were significantly enriched for two previously described IL-4 gene expression signatures [31, 32] (Fig. 1C). The top differentially expressed genes included known IL-4 regulated genes such as *CCL17*, *CCL22*, and *FCER2* (Fig. 1D), which encodes for the low-affinity cell surface receptor for IgE (FcεRII), alias CD23.

We validated increased *FCER2* gene expression in *STAT6*^MUT^ FL in another 138 diagnostic biopsies from the GLSG cohort with known mutation profile and available digital multiplexed gene expression data (nCounter, NanoString) (Fig. 1E). To validate enhanced CD23 protein expression and STAT6 pathway activation in *STAT6*^MUT^ FL, we performed immunohistochemistry (IHC) of primary patient samples. We indeed observed increased CD23 expression in *STAT6*^MUT^ FL compared to *STAT6*^WT^ FL (Fig. 1F, *left panel*). Moreover, we confirmed that pSTAT6 positive lymphoma cells formed prominent clusters in *STAT6*^MUT^ FL in the vicinity of IL-4 producing T_FH_ cells (Fig. 1F, *right panel*) as previously described [33].

### DNA binding site mutations are gain-of-function and require IL-4 for increased STAT6 activation

**Fig. 2:**
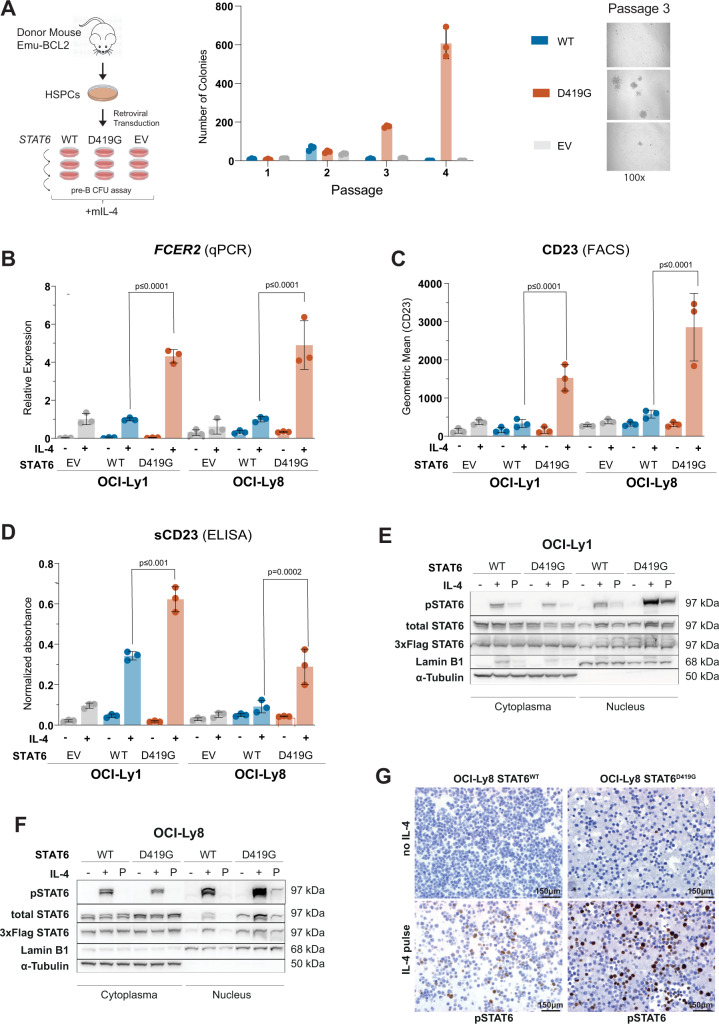
*STAT6* DNA binding site mutations are gain-of-function and require IL-4 for increased STAT6 activation. **A** Hematopoietic stem and progenitor cells (HSPCs) from Emu-BCL2 mouse cells transduced with either *STAT6*^WT^, *STAT6*^D419G^ or empty vector (EV) and serially replated on cytokine-supplemented methylcellulose (MethoCult, M3630) that supports the growth of mouse pre-B colony-forming units (CFUs) plus additional mouse IL-4 (mIL-4) (*N* = 3, mean ± SD). **B** *FCER2* mRNA levels (by qPCR) in OCI-Ly1 and OCI-Ly8 cells expressing either STAT6^WT^, STAT6^D419G^ or EV control after IL-4 stimulation (10 ng/mL, 24 h; 2^−dCt^ values relative to STAT6^WT^, *N* = 3, mean ± SD). **C** CD23 cell surface expression (by FACS) on OCI-Ly1 and OCI-Ly8 expressing either STAT6^WT^, STAT6^D419G^ or EV control after IL-4 stimulation (10 ng/mL, 24 h; geometric mean, *N* = 3, mean ± SD). **D** Soluble CD23 (sCD23) levels (by ELISA) in cell culture supernatants of OCI-Ly1 and OCI-Ly8 cells expressing either STAT6^WT^, STAT6^D419G^ or EV control after IL-4 stimulation (10 ng/mL, 72 h by ELISA (*N* = 3, mean ± SD). **E** Immunoblots of subcellular fractions (cytoplasmic vs nuclear) of OCI-Ly1 and **F** OCI-Ly8 cells expressing either STAT6^WT^ or STAT6^D419G^. “−“ indicates no IL-4 stimulation, “+” indicates IL-4 stimulation (10 ng/mL for 20 min), and “P” indicates IL-4 pulse stimulation (IL-4 10 ng/mL for 20 min, then wash & withdrawal of IL-4 and incubation for another 8 h in fresh media without IL-4). **G** Representative immunohistochemistry (IHC) stain for phosphorylated STAT6 (pSTAT6) in OCI-Ly1 cells expressing STAT6^WT^ or STAT6^D419G^ with or without IL-4 pulse stimulation.

To functionally test whether STAT6 DNA binding site mutations are gain-of-function, we utilized a modified pre-B colony-formation-unit (CFU) assay and analyzed the serial replating capacity. Specifically, bone marrow cells from mice expressing a BCL2-transgene in B-cells only (Emu-BCL2) were retrovirally transduced with *STAT6*^WT^, *STAT6*^D419G^ or EV control and plated on methylcellulose supplemented with cytokines that support the growth of pre-B CFUs plus additional mouse IL-4 (mIL-4). Beyond passage 3, only *STAT6*^D419G^ conferred a serial replating phenotype (Fig. 2A).

To study the molecular mechanisms of this IL-4 induced gain-of-function phenotype, we stably expressed STAT6^MUT^ (D419G, D419N, N421K, or D519V), STAT6^WT^ or EV control in OCI-Ly1 and OCI-Ly8 cells, two B cell lymphoma cell lines that harbor the FL hallmark translocation t(14;18). Upon IL-4 stimulation, we observed significantly increased *FCER2* gene expression in cells stably expressing STAT6^D419G^ (Fig. 2B), STAT6^D419N^, STAT6^N421K^, or STAT6^D519V^ (Supplementary Fig. 3A) as compared to STAT6^WT^. Likewise, IL-4 induced expression of CD23 on cell surfaces and soluble CD23 (sCD23) in cell supernatants was significantly increased in STAT6^MUT^ vs STAT6^WT^ cells (Fig. 2C, D). Importantly, we did not see any differences of STAT6^MUT^ vs STAT6^WT^ in the absence of IL-4 (Fig. 2B–D, Supplementary Fig. 3A–C).

### DNA binding site mutations promote aberrant IL-4 induced nuclear accumulation of pSTAT6

To study the mechanism of increased IL-4 induced transcriptional activation of STAT6^MUT^, we determined pSTAT6 levels in cytoplasmic and nuclear fractions. Following IL-4 stimulation, we observed higher nuclear pSTAT6 levels in cells expressing STAT6^D419G^ (Fig. 2E, F) or STAT6^D419N^ (Supplementary Fig. 4A) as compared to STAT6^WT^. Accordingly, cytoplasmic fractions were more depleted of pSTAT6 in STAT6^MUT^ vs STAT6^WT^ cells. Total STAT6 and pSTAT6 levels in whole cell lysates were not different (Supplementary Fig. 4B, C).

To determine the STAT6 signaling kinetics, we stimulated OCI-Ly1 and OCI-Ly8 cells with IL-4 for 20 min only, and then cultured the cells for an additional 8 h in the absence of IL-4 (“pulse stimulation” (P)), modeling the transient exposure of FL cells to IL-4 within the dynamic TME. Following IL-4 pulse stimulation, we detected higher levels of nuclear pSTAT6 in *STAT6*^MUT^ cells as compared to STAT6^WT^ cells (Fig. 2E, F, Supplementary Fig. 4C). Similar results were obtained when these cells were analyzed for pSTAT6 by IHC (Fig. 2G). IL-4 pulse simulation also led to significantly enhanced expression of *FCER2* mRNA and membrane-bound CD23 in STAT6^MUT^ vs STAT6^WT^ cells (Supplementary Fig. 3D, E). Thus, DNA binding site mutations in STAT6 lead to increased accumulation of pSTAT6 within the nucleus, and increased transcription and expression of STAT6 target genes.

### PARP14 is strongly upregulated in IL-4 stimulated STAT6^MUT^ cells

**Fig. 3:**
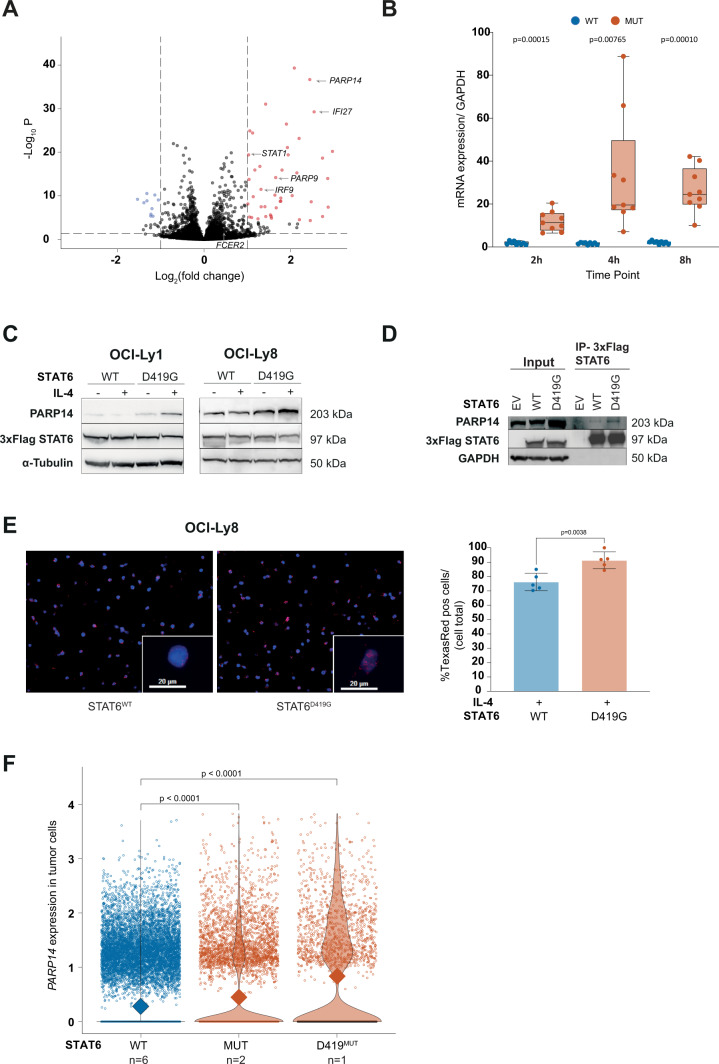
PARP14 is strongly upregulated in IL-4-stimulated *STAT6*^MUT^ lymphoma cells. **A** Volcano plot of differentially expressed genes from whole transcriptome sequencing of OCI-Ly1 cells expressing STAT6^WT^or STAT6^MUT^ (D419G, D419N, N421K) across all time points (i.e., 2, 4, and 8 h, respectively). Dashed lines indicate log_2_FC ±1.0, adj. *p* value 0.0001. **B** *PARP14* expression validated by quantitative PCR (qPCR) in OCI-Ly1 cells. **C** Immunoblot analysis of PARP14 protein expression in OCI-Ly1 and OCI-Ly8 expressing STAT6^WT^ or STAT6^D419G^ after IL-4 stimulation (10 ng/mL, 24 h). **D** Immunoprecipitation of 3xFlag-tagged STAT6 and immunoblotting for PARP14 and STAT6 (3xFlag) in OCI-LY8 after IL-4 stimulation (10 ng/mL, 24 h). **E** Proximity ligation assay (PLA) of STAT6 and PARP14. Representative images of OCI-Ly8 cells expressing STAT6^WT^ or STAT6^D419G^ after IL-4 stimulation (left); TexasRed channel used to detect red amplification signal. Box plots display the percentage of TexasRed positive cells per total cell number for each analyzed microscopic view field (*N* = 5, right). **F** Single-cell RNA sequencing analysis. Violin plot showing *PARP14* expression in tumor cells from 8 FL patients with wild type STAT6 (STAT6^WT^, *N* = 6) vs any STAT6 mutation (MUT, *N* = 2) vs STAT6 mutation at position D419 (D419^MUT^, *N* = 1).

We performed RNA sequencing of OCI-Ly1 cells expressing STAT6^MUT^ (D419G, D419N, or N421K, *N* = 3 each) or STAT6^WT^ (*N* = 9) at different time points following IL-4 stimulation (2, 4, and 8 h, respectively; Supplementary Fig. 5A). We selected OCI-Ly1 cells because of their marked responsiveness to IL-4. Principal components analysis revealed tight clustering of STAT6^D419G^, STAT6^D419N^, and STAT6^N421K^, mirroring the highly similar gain-of-function phenotype across the different mutations. This justified pooling of data from all STAT6^MUT^ cells for comparative analyses. In contrast, STAT6^WT^ was clearly separated from STAT6^MUT^ by PC2 (Supplementary Fig. 5B). Transcriptional changes over time were mostly represented by shifts in PC1 (Supplementary Fig. 5B). The expression levels of 54 genes were significantly different across all analyzed time points. Differential gene expression was skewed toward upregulation in STAT6^MUT^ vs STAT6^WT^, both by fold change and number of genes (Fig. 3A). Supplementary Fig. 5C shows the top differentially expressed genes for each time point and unsupervised clustering robustly separated STAT6^MUT^ from STAT6^WT^ cells. Reassuringly, we found *FCER2* to be differentially upregulated at the 8 h time point (log2 fold change 0.65, *p* = 0.0007). Other known STAT6 target genes such as *IRF4* and *SOCS1* were also significantly upregulated in STAT6^MUT^ vs STAT6^WT^ cells at one or more time points. Of note, *Poly(ADP-Ribose) Polymerase Family Member 14* (*PARP14*) was among the top most differentially upregulated genes across all time points (Fig. 3A, Supplementary Fig. 5C). We confirmed that IL-4 induced increased *PARP14* expression by qPCR (Fig. 3B) and by Western blot (Fig. 3C) in STAT6^MUT^ cells, whereas levels were not different in STAT6^WT^ cells. PARP14 has been reported to function as a transcriptional switch for STAT6-dependent gene activation [16]. Specifically, in the presence of IL-4 the catalytic activity of PARP14 facilitates STAT6 binding to the promoter, and release of transcriptionally repressive HDACs. We could indeed confirm direct interaction of PARP14 and STAT6 in IL-4 stimulated lymphoma cells by IP of 3xFlag-tagged STAT6 and immunoblotting for PARP14 (Fig. 3D). Furthermore, we performed proximity ligation assays (PLA) in OCI-Ly8 cells and showed significantly increased STAT6-PARP14 interaction upon IL-4 stimulation (Supplementary Fig. 6) and in IL-4 stimulated STAT6^D419G^ cells as compared to STAT6^WT^ cells (Fig. 3E).

### PARP14 is significantly upregulated in *STAT6*^MUT^ tumor cells in human FL

To test whether that *PARP14* is indeed upregulated in STAT6^MUT^ human FL, we analyzed single cell RNA sequencing data from human FL biopsies with known mutation status (*N* = 8, Supplementary Fig. 7) from Haebe et al. [28]. In fact, tumor cells from FLs harboring any *STAT6*^MUT^ (*N* = 2), and *STAT6*^D419G^ in particular (*N* = 1), had significantly higher *PARP14* expression compared to *STAT6*^WT^ tumor cells (Fig. 3F). Of note, this difference in *PARP14* expression was only seen in a subpopulation of tumor cells, consistent with our hypothesis that this phenotype is restricted to FL cells that harbor the *STAT6* mutation and are exposed to TME-derived IL-4, i.e., localized in spatial proximity to IL-4 producing T_FH_ cells.

### Validation in a human ex vivo FL-like co-culture system

**Fig. 4:**
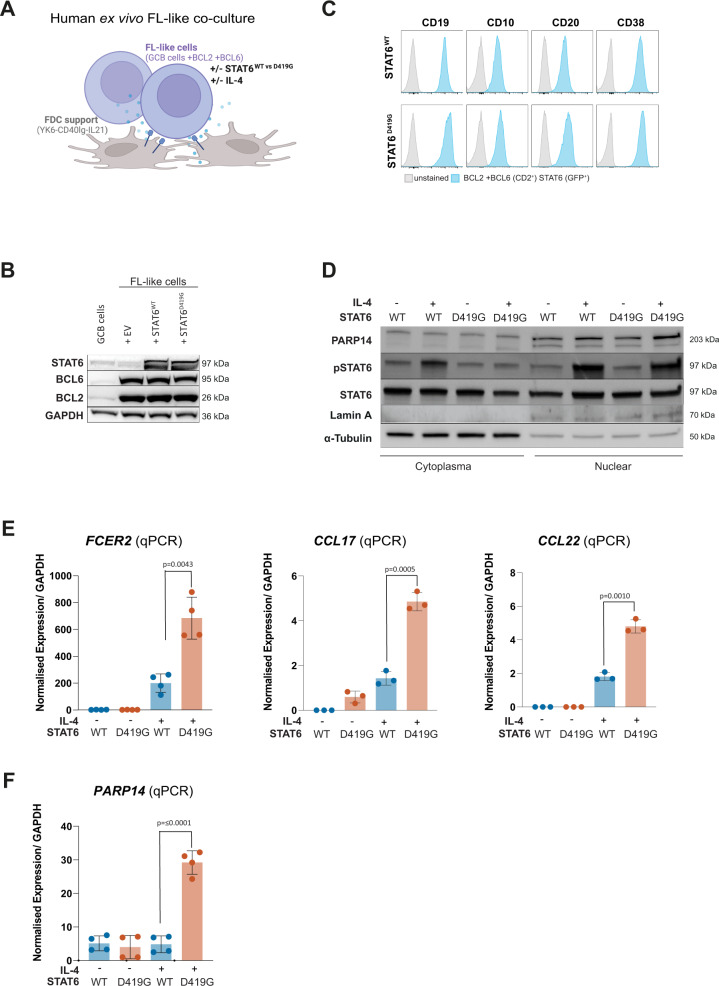
Validation experiments in a human ex vivo FL-like co-culture system. **A** Schematic of a fully human FL-like model system: human tonsil-derived germinal center (GC) B cells expressing BCL2 and BCL6 as well as STAT6^WT^ or STAT6^D419G^ or EV are co-cultured with follicular dendritic cell (FDC) feeder cells that express CD40L and IL-21 (YK6-CD40lg-IL-21). **B** Western Blot of FL-like cells, whole cell lysates. **C** Flow cytometry of GC markers on FL-like cells with indicated *STAT6* genotypes. **D** Western Blot of FL-like cells, cellular and nuclear fraction. **E** qPCR analysis of *FCER2*, CCL17, and CCL21, as well as **F** *PARP14* in FL-like cells with indicated *STAT6* genotypes with and without IL-4 stimulation.

We wanted to validate our findings in an ex vivo model that more closely resembles human FL. Therefore, we immortalized human tonsil-derived GC B cells by stably expressing BCL2 and BCL6, along with either STAT6^WT^, STAT6^D419G^ or EV (Fig. 4A, B). Importantly, these cells maintain a GC phenotype (Fig. 4C) and absolutely require FDC support plus IL21 and CD40L (YK6-CD40lg-IL21) for sustained growth, mirroring the TME-dependence of FL.

Using this FL-like co-culture we could indeed confirm our previous findings, including increased IL-4 induced nuclear levels of pSTAT6 and PARP14 in STAT6^D419G^ cells compared to STAT6^WT^ cells (Fig. 4D), significantly increased IL-4 induced gene expression of the known STAT6 target genes *FCER2*, *CCL17*, and *CCL22* (Fig. 4E), as well as significantly increased *PARP14* expression only in IL-4 stimulated STAT6^D419G^ cells (Fig. 4F).

### PARP14 per se is a novel target of mutant STAT6

**Fig. 5:**
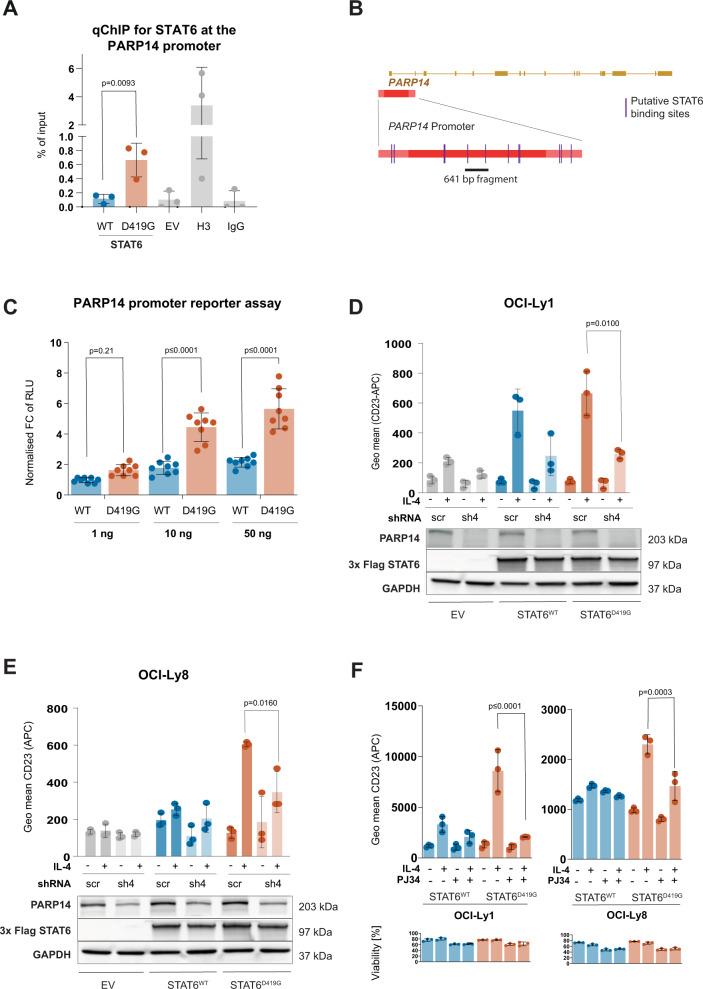
PARP14 per se is a target gene of mutant (STAT6^MUT^) but not wild type STAT6 (STAT6^WT^). **A** Cross-linked chromatin immunoprecipitation (ChIP) of 3xFlag-tagged STAT6^WT^ or STAT6^D419G^ in OCI-LY8 cells after IL-4 stimulation (10 ng/mL, 24 h) followed by quantitative PCR for the PARP14 promoter region (qChIP). **B** Schematic of the PARP14 promotor region, indicating predicted / putative STAT6 binding sites and the 641 bp region that was cloned into pGL3. **C** PARP14 promoter luciferase assay (pGL3) with increasing amounts of co-transfected *STAT6*^WT^ or *STAT6*^D419G^ in 293 T cells in the presence of IL-4 (24 h after transfection, 10 ng/mL for 6 h). Shown are fold changes (FC) of luciferase activity (relative light unit, RLU) normalized to 1 ng STAT6^WT^ (*N* = 6, mean ± SD). **D** CD23 cell surface expression (by FACS) on OCI-Ly1 and **E** OCI-Ly8, each expressing either STAT6^WT^ or STAT6^D419G^ with shRNA-mediated knock-down of PARP14 (sh4) or a non-targeting (scrambled) control (scr), respectively. Immunoblot of respective cells as indicated below. **F** CD23 cell surface expression by FACS on OCI-Ly1 and OCI-L8 expressing STAT6^WT^ or *STAT6*^*D*419G^ with or without IL-4 stimulation (10 mg/mL, 24 h) and treatment with the PARP inhibitor PJ34 (50 µM, 15 min prior to IL-4 stimulation) or vehicle, respectively. (*N* = 3, mean ± SD). Cell viability of cells as indicated below (*N* = 3, mean ± SD).

We hypothesized that PARP14 per se could be a novel (aberrant) target gene of STAT6^MUT^, but not of STAT6^WT^. To directly assess and quantify its binding to the PARP14 promotor, we performed cross-linked chromatin IP for 3x Flag of IL-4 stimulated lymphoma cells (OCI-Ly8) stably expressing either 3x Flag-tagged STAT6^MUT^ (D419G) or 3xFlag-tagged STAT6^WT^ followed by quantitative PCR for the PARP14 promotor region. This indeed demonstrated increased binding of STAT6^D419G^ to the PARP14 promoter, whereas STAT6^WT^ was not different compared to EV control (Fig. 5A). We identified several putative STAT6 binding sites in the PARP14 promotor by bioinformatic prediction (Fig. 5B). To functionally validate our finding of increased binding of STAT6^MUT^ to the PARP14 promotor, we cloned a 621 bp fragment containing two potential STAT6 binding sites in close proximity to the PARP14 start codon into a luciferase reporter construct. The reporter construct was then expressed in HEK 293 T cells, along with either STAT6^D419G^ or STAT6^WT^. We used HEK 293 T cells as they express the IL-4 receptor but no endogenous STAT6. In the presence of IL-4, STAT6^D419G^ indeed showed significantly increased transactivation activity compared to STAT6^WT^ (Fig. 5C) in a dose-dependent manner.

### Inhibition of PARP blocks the mutant STAT6 gain-of-function phenotype

We wanted to exploit the potential of targeting PARP14 to block the STAT6^MUT^ phenotype. Since 293T cells do not express endogenous PARP14 we used HeLa cells. We first expressed two different shRNAs to knock-down PARP14 and again quantified STAT6-activated transcriptional activity by luciferase assay. In fact, knock-down of PARP14 significantly reduced transactivation activity of both STAT6^D419G^ and STAT6^D419N^, correlating with the efficiency of PARP14 knock-down (Supplementary Fig. 8). In contrast, STAT6^WT^ showed no baseline transcriptional activity and was unaffected by PARP14 knock-down (Supplementary Fig. 8).

Next, we knocked-down PARP14 in OCI-Ly1 and OCI-Ly8 stably expressing either *STAT6*^WT^, *STAT6*^D419G^ or EV. In both cell lines shRNA-mediated knock-down of PARP14 (sh4) resulted in significant reduction of IL-4 induced CD23 expression compared to scrambled control (scr) and completely abrogated the STAT6^MUT^ gain-of-function phenotype (Fig. 5D, E).

Finally, we sought to inhibit PARP14 pharmacologically. For this, OCI-Ly1 and OCI-Ly8 cells stably expressing STAT6^MUT^ or STAT6^WT^ were treated with the PARP inhibitor PJ34, in the absence or presence of IL-4, and assayed for STAT6 activation by CD23 expression. PARP inhibition completely blocked IL-4 induced expression of CD23 in cells expressing STAT6^D419G^ down to levels observed with STAT6^WT^ (Fig. 5F). Vice versa, we did not observe a significant effect of PJ34 treatment in the absence of IL-4 or in cells expressing STAT6^WT^.

## Discussion

Re-education of the TME has been identified as a hallmark of FL, actually enabling its pathogenesis and progression [34]. Furthermore, the composition of the TME has been shown to impact clinical outcome of patients with FL [10, 11, 35, 36]. However, the molecular mechanisms that orchestrate and maintain this re-education process in genetically defined subsets of FL often remain elusive, yet they hold great potential as therapeutic targets.

**Fig. 6:**
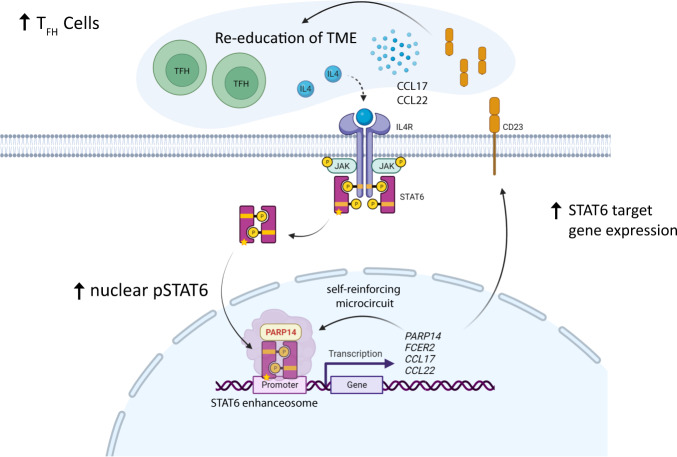
Model of a PARP14-mediated self-reinforcing regulatory circuit that amplifies IL-4 induced transcriptional activity in STAT6^MUT^ FL. *STAT6* mutations amplify IL-4 induced STAT6-dependent gene activation via an intracellular self-reinforcing regulatory microcircuit that involves aberrantly increased PARP14 levels in IL-4 stimulated STAT6^MUT^ FL cells. Increased STAT6-dependent gene expression involves cytokines (e.g., CCL17 and CCL22) which contribute to the re-education of the tumor microenvironment, including increased recruitment of IL-4 producing T follicular helper (T_FH_) cells. Details see text. Yellow star indicates STAT6 mutation. Figure created with BioRender.com.

Here, we add novel and clinically relevant aspects to seminal studies that established T_FH_-derived IL-4 [13, 37, 38] and *STAT6* mutations [23] as drivers in FL. Specifically, we describe a therapeutically targetable PARP14-mediated self-reinforcing regulatory circuit that amplifies IL-4 induced transcriptional activity of STAT6^MUT^ (Fig. 6).

Conceptually, we propose that this IL-4 driven re-education process of the TME is composed of at least two self-reinforcing regulatory circuits, an intracellular and an extracellular route (Fig. 6). The extracellular route has been well described and involves cytokines like CCL17 and CCL22 in the FL TME [39]. Both chemokines are produced by FL cells in response to IL-4 stimulation (and CD40L) and promote recruitment of Tregs and further accumulation of IL-4 producing T_FH_ cells [39]. Our data shows that these cytokines are indeed among the top upregulated genes in primary patient biopsies of *STAT6*^MUT^ FL, supporting the concept that *STAT6* mutations amplify the IL-4 driven re-education process of the FL TME. In this work, we focused on the molecular mechanisms of the intracellular route (the microcircuit) and identified several aspects of clinical-translational relevance.

First, the gain-of-function phenotype of STAT6^MUT^ was strictly dependent on the presence of IL-4 stimulation. Hence, the biological and clinical relevance of *STAT6* mutations in patients may only be adequately evaluable in the context of the TME composition, specifically including T_FH_ abundance and distribution and IL-4 levels. This could explain why the *STAT6* mutation status per se has not yet been found to be associated with differences in the clinical course of the disease or treatment outcome [21]. In fact, when we performed an exploratory analysis of our previously reported cohort of patients, who received standard immunochemotherapies (R-CHOP or R-CVP) for advanced stage FL [21], we observed only a trend toward shorter failure-free survival for patients with *STAT6*^MUT^ FL, but this did not reach statistical difference (data not shown). We hypothesize that combined biomarkers, e.g., integrating the T_FH_ abundance/TME composition and/or IL-4 levels with *STAT6* mutation status, will help to further refine patient stratification [36].

Interestingly, an alternative mechanism of constitutive IL-4 signaling and STAT6 activation independent of external IL-4 stimulation has been reported for another B cell lymphoma subtype, primary mediastinal B cell lymphoma (PMBL). Specifically, up to a quarter of PMBL harbor gain-of-function mutations in the IL-4R alpha chain, which, of note, frequently co-occur with additional mutations in the JAK/STAT pathway, including *STAT6* [40], further supporting the concept that *STAT6* mutations function primarily as amplifiers of this signaling cascade. While we did not find *IL-4R* mutations in our previously reported cohort of patients with untreated FL (*N* = 112, GLSG2000 cohort) [21], this may have to be re-addressed in larger cohorts including patients with variant histologies that indicate less dependence on the TME, such as diffuse FL or histologically transformed FL [18–20, 41, 42].

Furthermore, we notice that STAT6^D419N^, which is annotated as a missense germline polymorphism (rs11172102) exerts a gain-of-function phenotype similar to all other somatic STAT6 mutations affecting the DNA binding domain. In the absence of matched germline control, we cannot ultimately distinguish between rare germline polymorphisms and somatically acquired mutations in each of these cases. Importantly however, irrespective of its acquisition, our data indicates that this amino acid change in the DNA binding domain similarly contributes to the distinct biology of *STAT6*^MUT^ FL. Thus, caution should be taken when sequencing data is analyzed using standard pipelines, which frequently filter out putative or confirmed germline variants.

Finally and importantly, we identified PARP14 as a novel STAT6^MUT^-specific target gene. PARP14 was previously found to associate with STAT6 [43] and function as a transcriptional switch and activator of STAT6-dependent gene expression in the presence of IL-4 [44]. Our data support a model in which increased PARP14 levels drive a self-reinforcing microcircuit that promotes the assembly of the STAT6 enhanceosome complex in IL-4 stimulated *STAT6*^MUT^ lymphoma cells, thereby further amplifying STAT6-dependent gene activation. Of note, the catalytic activity of PARP14 has been shown to be essential for the transcriptional enhancement function of PARP14 [15]. In fact, we show that pharmacological inhibition of the PARP enzymatic activity was able to completely abrogate the STAT6^MUT^ gain-of-function phenotype.

Our study also raises some interesting successive hypotheses that should be addressed in future studies. It is, for instance, intriguing to speculate that increased *PARP14* levels may have additional functional consequences. As such, PARP14 has been implicated in mediating IL-4 induced attenuation of caspase-3 activation [45], i.e., increased PARP14 levels could contribute to protection against apoptosis and provide a survival advantage to STAT6^MUT^ lymphoma cells. Furthermore, our study provides proof-of-principle that the identification and functional characterization of aberrant targets of STAT6^MUT^, potentially not limited to PARP14, as well as aberrant targets of other recurrently mutated transcription factors holds promise for developing individualized treatment strategies.

In summary, we show that STAT6 mutations amplify IL-4 induced STAT6-dependent gene activation via a self-reinforcing regulatory circuit that involves aberrantly increased PARP14 levels, and therefore represents a novel therapeutic target in STAT6^MUT^ FL.

## Supplementary information

Supplement-clean version

SuppFig.1

SuppFig.2

SuppFig.3

SuppFig.4

SuppFig.5

SuppFig.6

SuppFig.7

SuppFig.8
