# Supplementary material for: PARP14 is a novel target in STAT6 mutant follicular lymphoma
Source: Leukemia. 2022 Jul 18;36(9):2281–92. doi: 10.1038/s41375-022-01641-x (PMC9417990; doi:10.1038/s41375-022-01641-x)
Supplement: Supplementary file 1 — Supplement-clean version [file 41375_2022_1641_MOESM1_ESM.docx]

**Supplementary Data: PARP14 is a Novel Target in *STAT6* Mutant Follicular Lymphoma**

**Table of contents**

[**Supplementary Methods** 2](#_Toc104733421)

[**Supplementary Tables** 9](#_Toc104733422)

[**Supplementary References** 11](#_Toc104733423)

[**Supplementary Figure Legends** 12](#_Toc104733424)

# **Supplementary Methods**

**Patient cohort:**

The clinical, gene mutation and genome-wide gene expression data (GSE66166) were derived from previously reported patient cohorts from the *German Lymphoma Study Group* (GLSG) and *British Columbia Cancer Agency* (BCCA) (1).

**Gene set enrichment analysis (GSEA):**

GSEA was performed on gene expression data from 106 BCCA patients using the desktop application (v3.0) from the Broad Institute (2, 3). We compared *STAT6*^WT^ and *STAT6*^MUT^ cases. 1000 permutations were performed to assess the statistical significance of the enrichment score. Permutation type was set to phenotype. For all other parameters default values were used. We tested for the previously described IL-4 gene expression signatures LU_IL4_SIGNALING and PID_IL4_2PATHWAY (4, 5).

**Immunohistochemistry:**

For formalin-fixed paraffin-embedded FFPE blocks, cell lines were fixed in 4.5% formalin solution, mixed with isopropanol and glycerin. The pellet was embedded using standard procedures for tissues. For immunohistochemical staining of tissue sections or FFPE-embedded cells, the following staining procedures were applied:

- anti-CD23 (Novocastra, Leica Biosystems, Nußloch, Germany, Clone 1B12, dilution 1:20): Leica BOND automated stainer, antigen retrieval program ER1.
- PE Mouse Anti-Stat6 (pY641) (BD Biosciences, Franklin Lakes, New Jersey USA, Clone 18, dilution 1:1000): Heat-induced antigen retrieval with pressure cooker (3 min, citrate buffer pH 6.0). Manual staining using CSA-Kit (Dako, Glostrup, Denmark).

**STAT6 constructs:**

The STAT6 plasmid was purchased from Harvard Plasmid repository (HsCD00365550) and tagged with C-terminal 3xFlag. DNA binding site mutations were introduced by site-directed mutagenesis using Phusion High-Fidelity polymerase (New England Biolabs, [Ipswich, Massachusetts](https://www.google.com/search?sxsrf=AOaemvKNkqEUe-DU5L9EZS59-jsJtXzaQQ:1643287275666&q=Ipswich,+Massachusetts&stick=H4sIAAAAAAAAAONgVuLUz9U3MMw1L6h8xGjCLfDyxz1hKe1Ja05eY1Tl4grOyC93zSvJLKkUEudig7J4pbi5ELp4FrGKeRYUl2cmZ-go-CYWFycmZ5QWp5aUFAMANKWI510AAAA&sa=X&ved=2ahUKEwirpdGS-tH1AhXX8rsIHYKtCq0QzIcDKAB6BAgWEAE) USA). The STAT6 constructs were cloned into the CMV-driven expression vector pHAGE-CMV-MCS-IRES-ZsGreen (Harvard PlasmID, EvNO00061605) or into pMSCV-IRES-GFP (Addgene 52107, Watertown, Massachusetts, USA) by Gateway cloning (Invitrogen, [Waltham, Massachusetts](https://www.google.com/search?biw=1172&bih=645&sxsrf=AOaemvJfyt2pGFg3VSYngNW17OJtF5J8xw:1643287823916&q=Waltham&stick=H4sIAAAAAAAAAONgVuLQz9U3yKgyNn3EaMwt8PLHPWEprUlrTl5jVOHiCs7IL3fNK8ksqRQS42KDsnikuLjgmngWsbKHJ-aUZCTmAgDLoZJyTAAAAA&sa=X&ved=2ahUKEwi1yYeY_NH1AhVv77sIHeRlBjsQzIcDKAB6BAgQEAE), USA). Primers used for cloning are listed in **Supplementary Table 1**.

**Flow cytometry:**

Cells were stimulated with IL-4 (10 ng/mL, Miltenyi Biotec, Cologne, Germany) for 24 hours. In experiments which included use of PARP inhibitor PJ34 (Selleckchem, Munich, Germnay), cells were treated with 50 µM inhibitor 30 minutes prior to IL-4 stimulation. Cells were stained with anti-CD23 (BD Biosciences, Clone M-L233,APC, 1:50) and DAPI (Promocell, Heidelberg, Germany).

**Quantitative real time PCR (qPCR):**

Cells were stimulated with IL-4 (10 ng/mL) for either 24 hours or 20 minutes, followed by 1x wash with PBS and a further incubation for 8 hours. Total RNA was isolated using TRIzol^®^ reagent (Ambion, Austin, Texas, USA). Synthesis of complementary DNA was performed with SuperScript^®^ III First-Strand Synthesis System (Invitrogen,). *FCER2*, *PARP14* and *GAPDH* transcripts were quantified using the Fast SYBR Green Master Mix (Applied Biosystems, [Waltham, Massachusetts](https://www.google.com/search?biw=1172&bih=645&sxsrf=AOaemvJfyt2pGFg3VSYngNW17OJtF5J8xw:1643287823916&q=Waltham&stick=H4sIAAAAAAAAAONgVuLQz9U3yKgyNn3EaMwt8PLHPWEprUlrTl5jVOHiCs7IL3fNK8ksqRQS42KDsnikuLjgmngWsbKHJ-aUZCTmAgDLoZJyTAAAAA&sa=X&ved=2ahUKEwi1yYeY_NH1AhVv77sIHeRlBjsQzIcDKAB6BAgQEAE), USA) on a 7900HT Fast Real-Time PCR System (Applied Biosystems). Primers used for qPCR are listed in **Supplementary Table 1**.

**ELISA for soluble CD23:**

Cells were stimulated with IL-4 (10 ng/mL) for 48 hours. Cell culture supernatants were filtered through a 0.22 µm syringe filter to remove cell debris. Supernatants were assayed with the CD23 (soluble) Human ELISA Kit (Invitrogen), according to the manufacturer’s protocol. Absorbance values were corrected by viable cell count.

**Immunoblot analysis:**

1x10^7^ cells were lysed using radio immunoprecipitation assay buffer (RIPA) (Thermo Fisher Scientific, Waltham, Massachusetts, USA). Protein concentrations were quantified using the Pierce BCA assay (Thermo Fisher Scientific) and measured on a GloMax plate reader (Promega, Madison, Wisconsin, USA). Proteins were separated on 4-12% SDS-PAGE (Invitrogen). Immunoblots were transferred using the X Cell II Blot module (Thermo Fisher Scientific), blocked for 1 hour and then incubated with primary antibodies overnight, 4°C. Blots were washed 3x 10 minutes and incubated with secondary horseradish radish peroxidase antibody for 1 hour and then subsequently washed as before. Blots were developed using Pierce ECL blotting substrate (Thermo Fisher Scientific). Subcellular fractions were made using Qproteome Nuclear Protein Kit (Qiagen). Antibodies used for western blot analysis are listed in **Supplementary Table 2**.

**Immunoprecipitation:**

STAT6^WT^, STAT6^D419G^ or EV expressing cells were stimulated with IL-4 (10 ng/mL) for 24 hours. Cells were lysed using passive lysis buffer (PLB, Promega), supplemented with proteinase inhibitor (Sigma Aldrich, St. Louis, Missouri, USA)) for 30 minutes on ice, followed by centrifugation (30 minutes at 15,596 x g). 2.5 mg of isolated protein was mixed in a total volume of 5 mL PLB. 50μl of M2 Flag agarose bead slurry (Sigma Aldrich) was added to the lysate and incubated overnight at 4°C. Samples were then washed by incubation with PLB for 5 minutes rotating (50 rpm) and then centrifugation (30 seconds, 800 x g). This was repeated three times. Subsequently, 10x SDS (Invitrogen) was added to the sample to elute the protein. Samples were rotated 10 minutes at room temperature. The eluted protein was then removed from the beads via centrifugation (5 min, 800 x g) and the elute heated at 95°C for 5 minutes. Input and eluted protein samples were then analyzed via western blotting as described.

**Pre-B CFU assay:**

Emu BCL2 mice (B6.Cg-Tg(BCL2)36Wehi/J) were obtained from Jackson Laboratory. Mice were sacrificed at 6-10 weeks of age using isoflurane and cervical dislocation. Pre-B CFU assay was performed as described in the main manuscript.

**RNA sequencing:**

For differential gene expression analysis *STAT6* D419G, D419N, and N421K data was combined (*STAT6*^MUT^) and compared to *STAT6*^WT^. Data analysis was performed for across all time points and for each time point, respectively. The FDR threshold was set to 5%. Differential expression was statistically verified by Wald test and corrected for multiple testing using the Benjamini-Hochberg adjustment. Data was visualized with *pheatmap* (v1.0.8) and *ggplot2* (v2.2.1) packages (6).

**Single cell RNA sequencing:**

Single cell RNA sequencing (scRNA-seq) from one tumor site and hybrid capture DNA sequencing data for 8 patients with follicular lymphoma were derived from a prior published study (7). Standard scRNA-seq analysis (normalization, scaling and clustering) using the R package Seurat (version 3) (Stuart, Butler et al, Cell 2019), cell cluster annotation, and classification of B cells into malignant and non-malignant clusters were performed as previously described (7). Of note, in comparison to the prior study, a resolution of 1 for shared nearest neighbor (SNN)-based clustering and for Uniform Manifold Approximation and Projection (UMAP)-based visualization was used. Differential expression analysis was performed using the Wilcoxon Rank Sum test.

**Proximity Ligation Assay (PLA):**

OCI-Ly8 cell lines stably expressing STAT6^WT^ or STAT6^D419G^ were either left untreated or treated with 10ng/mL IL-4 for 24 hours. A total of 10 x 10^6^ cells were harvested, washed with PBS twice and fixed with 4% paraformaldehyde at 4°C overnight before they were embedded in paraffin according to standard protocols for tissue preparation. Two μm tissue sections were deparaffinized in xylene and rehydrated in graded alcohol and distilled water. Heat induced antigen retrieval was performed on tissue sections at pH 9.0 for 30 minutes (AR9 buffer, Akoya).

For PLA, slides were blocked in Duolink Blocking buffer (Sigma) for 60 min at 37°C. Cells were co-stained with mouse anti-STAT6 (LSBio, LS-B6154, 1:800) and rabbit anti-PARP14 (Sigma, HPA012063, 1:100). Primary antibodies were diluted in Dako Antibody Diluent (Dako) and incubated for 60min at room temperature. Cells were washed for 10 min in Duolink Wash Buffer A (Sigma), followed by addition of the appropriate Duolink secondary antibodies (PLA-Probe Mouse MINUS, PLA-Probe Rabbit PLUS, Sigma), diluted and mixed according to the manufacturer’s instructions. Cells were incubated for 1 hour at 37 °C. Cells were washed in Duolink Wash Buffer A for 10 min. Ligation and amplification steps of the PLA were performed using the Duolink in situ Detection Reagents Red kit (Sigma) according to the manufacturer’s instructions for 30 min and 100 min respectively. Following the PLA, cells were stained with DAPI for 5min at RT and mounted in Prolong Diamond mounting media (ThermoFisher).

Images were acquired on a Vectra Polaris imaging system using inForm Automated Image Analysis Software (Akoya) for display. PLA spots (TexasRed channel) per cell were counted in five representative view fields (40x) per cell clone and treatment using HALO Image Analysis Platform version 3.2. Box plots display the percentage of TexasRed positive cells per total cell number for each analyzed microscopic view field (N = 5).

**Quantitative chromatin immunoprecipitation (qChIP):**

qChIP was performed as previously described (8) with the following specifics: 12 x 10^6^ STAT6^WT^, STAT6^D419G^ or EV cells were seeded and stimulated with IL-4 (10 ng/mL) for 24 hours. Cells were washed and resuspended in PBS (1 mL volume). EGS crosslinker (Sigma Aldrich) was then added to the cells for 30 minutes (1.5 mM), lightly shaking. 1% formaldehyde (Thermo fisher Scientific) was additionally added for 10 minutes before decrosslinking with 0.125 M glycine (Sigma Aldrich) for 5 minutes. Samples were centrifuged (5 minutes, 4000 rpm) and 1 mL cell lysis buffer was added for 10 minutes on ice. SDS buffer was then added for a further 10 minutes on ice before sonication. Sonication was performed on a Bioruptor Pico (Diagenode, for 20 minutes. Sonication was verified on a 1% agarose gel. Sheared chromatin was quantified from the input and 15 µg was used per chromatin immunoprecipitation. Samples were diluted and the input samples removed. STAT6 (1:50) or control antibodies were added according to the manufacturer’s recommendations, and the samples incubated overnight. ChIP grade magnetic beads (Cell Signaling, Danvers [Massachusetts,](https://www.google.com/search?q=Danvers,+Massachusetts&stick=H4sIAAAAAAAAAONgVuLUz9U3MMypKM97xGjCLfDyxz1hKe1Ja05eY1Tl4grOyC93zSvJLKkUEudig7J4pbi5ELp4FrGKuSTmlaUWFeso-CYWFycmZ5QWp5aUFAMAcCMVc10AAAA&sa=X&ved=2ahUKEwie8Ynw8tf1AhUdSPEDHeCWDqQQzIcDKAB6BAgREAE) USA) were added to each IP according to manufacturer instructions. Following a 3-hour incubation, magnetic beads were isolated and washed in high and low salt buffers, lithium chloride (LICL) and (Tris-HCL-EDTA) TE (2x). Subsequently IPs were incubated with SDS buffer for 15 minutes twice, each time collecting the supernatant. All samples were then de-crosslinked via adding NaCl (0.2M, Sigma Aldrich) overnight (62°C, 500rpm). Samples were then treated with RNAse (0.5mg/mL, Qiagen, 37°C for 2 hours) and Proteinase K (0.1mg/mL, Bioline, Toronto, Ontario, Canada) overnight (42°C) and the DNA was isolated using a PCR purification kit (Qiagen). SYBR green qPCR (Invitrogen) was performed as described in (8). All buffers were prepared according to a previous publication (8). Antibodies for qChIP are listed in **Table 3**.

**Luciferase reporter assay construct:**

A 621 bp fragment from the PARP14 promoter (ENSR00000305844) was cloned from OCI-Ly8 gDNA and ligated into pGL3-Basic luciferase reporter vector (Promega). It contained two STAT6 binding sites (highlighted in bold), identified by MatInspector software (Genomatix (9).

chr3: 122,680,699-122,681,381 (Assembly GRCh38.p12)

GGATGACTCTGCCATTCCTGggtccgctgtgttaggctgaaggcaaaaggaaaccaaggcctgggagtt**ttccaggaa**acgaaagcgaaagagtcaaagttagcggcccggagttggcgcggcccctgcagtccggcggagagcggagctgaggatggctgtgcccggctccttcccgctgctggtcgagggctcctggggccccgaccccccgaagaacttgaacaccaagttgcagatgtacttccagagcccgaagaggtcgggaggcggcgagtgtgaggtccgccaggatcccaggagcccatcccgcttcctggtgttcttctacccggaggacggtgaggggcgcgaggggtggggtgaggagggggcacctctgccctccctccagggaaatggcggcagggcacgcacgggagggtgacccgcccgacttcggcggctgctgtagcggaggtggccggggcgggggcgggggcgggggcggcagaatggattccgagcgcacccggggcgctgcggttccccggcgccctgcgc**ttccagg**cgcttaatggcgcggcccggaggtggcggcggaaccgcgcaagtaactCTTTATCCCTCGATCGTTTTCTG

**PARP14 knockdown:**

PARP14 knockdown constructs were purchased from Sigma Aldrich and are listed in **Supplementary Table 4**.

**Statistical Analysis:**

Data from at least three independent experiments was collected and is expressed as mean value ± SD. To assess weather differences between groups were significant, Welch's t-test or two-way ANOVA was performed using GraphPad Prism software (version 6.07). Multiplicity adjusted p-values by Dunnett's multiple comparisons test are reported. Statistical significance was defined as p<0.05. Sample size of each experiment is listed in the respective figure and/or figure legend.

# **Supplementary Tables**

**Suppl. Table 1- Primers used in this study**

| **Application** | **Sequence (5’ to 3’)** |
| --- | --- |
| **Cloning:** |  |
| Addition of attB site & Kozak sequence | G GGG ACA ACT TTG TAC AAA AAA GTT GGC CACC ATG TCT CTG TGG GGT CTG GTC TCC AAG |
| Addition of attB site, Stop codon, & 3x Flag sequence | GGG GAC AAC TTT GTA CAA GAA AGT TGG CTA CTT GTC ATC GTC ATC CTT GTA GTC GAT GTC ATG ATC TTT ATA ATC ACC GTC ATG GTC TTT GTA GTC CCA ACT GGG GTT GGC CCT TAG GTC C |
| Site-directed mutagenesis D419G FP | GGC AAC CAA GGC AAC AAT GCC AAA GCC AC |
| Site-directed mutagenesis D419G RP | CAT TGT TGC CTT GGT TGC CAT GGA CGA TG |
| Site-directed mutagenesis D419N FP | GGC AAC CAA AAC AAC AAT GCC AAA GCC AC |
| Site-directed mutagenesis D419N RP | CAT TGT TGT TTT GGT TGC CAT GGA CGA TG |
| Site-directed mutagenesis N421K FP | GAC AAC AAA GCC AAA GCC ACT ATC CTG TGG |
| Site-directed mutagenesis N421K RP | TGG CTT TGG CTT TGT TGT CTT GGT TGC CAT G |
| Site-directed mutagenesis D519V FP | GCA GTG GTT TGT TGG TGT CCT GGA CCT CAC |
| Site-directed mutagenesis D519V RP | AGG ACA CCA ACA AAC CAC TGC CAA AAG GTG AAG |
| **Quantitative PCR:** |  |
| GAPDH FP | CAC CCA CTC CTC CAC CTT TG |
| GAPDH RP | TCT CTC TCT TCC TCT TGT GCT CTT G |
| PARP14 FP | GAC TGT CGC TAT GTG CTT CAC |
| PARP14 RP | GGA CAA GCT CTC AGT GAT CTC C |
| **Luciferase reporter assay construct:** |  |
| PARP14_promoter FP | CCG CTC GAG GGA TGA CTC TGC CAT TCC TG |
| PARP14_promoter RP | CCC AAG CTT CAG AAA ACG ATC GAG GGA TAA AG |
| **qChIP** |  |
| PARP14 FP | TTT GTA AAG GGT CGG CTT GC |
| PARP14 RP | AGA TCA AGT CGG CAG CTT TG |

**Suppl. Table 2- Antibodies used for western blot analysis**

| **Name** | **Supplier** | **Clone** | **Product number** | **Dilution** |
| --- | --- | --- | --- | --- |
| Anti-Lamin B1 | Abcam | EPR8985(B) | ab133741 | 1:2500 |
| Anti-PARP14 | Sigma- Aldrich |  | HPA012063 | 1:250 |
| Anti-α-Tubulin | Sigma-Aldrich | DM1A | T6199 | 1:20 000 |
| Monoclonal ANTI-FLAG^®^ M2 | Sigma-Aldrich | M2 | F3165 | 1:2500 |
| Phospho-Stat6 (Tyr641) | Cell Signaling Technology | Polyclonal | 9361 | 1:2000 |
| STAT6 | Cell Signaling Technology | Polyclonal | 9362 | 1:2000 |
| GAPDH | Invitrogen | 6C5 | **AM4300** | 1:20 000 |

| **Name** | **Supplier** | **Clone** | **Product number** | **Dilution** |
| --- | --- | --- | --- | --- |
| STAT6 | Cell Signaling Technology | D3H4 | 5397S | 1:50 |
| Histone H3 | Cell Signaling Technology | Polyclonal | 2650S | 1:50 |
| IgG XP Isotype Control | Cell Signaling Technology | DA1E | 3900S | Lot dependent concentration of STAT6 |

**Suppl. Table 3- Quantitative chromatin immunoprecipitation (qChIP) antibodies**

| **Supplier/ Catalog number** | **Construct** | **Sequence** |
| --- | --- | --- |
| Sigma-Aldrich SHC016-1EA | MISSION pLKO.1-puro Non-Target shRNA Control | CCGGGCGCGATAGCGCTAATAATTTC  TCGAGAAATTATTAGCGCTATCGCGCTTTTT |
| Sigma-Aldrich TRCN0000053158 | MISSION R pLKO.1-puro PARP14 shRNA #4 | CCGGGCACCATTTGAAGAGTCACTAC  TCGAGTAGTGACTCTTCAAATGGTGCTTTTTG |
| Sigma-Aldrich TRCN0000053159 | MISSION R pLKO.1-puro PARP14 shRNA #5 | CCGGCGGAACTTCATTCTTCACAAA  CTCGAGTTTGTGAAGAATGAAGTTCCGTTTTTG |

**Suppl. Table 4- PARP14 shRNA plasmids**

# **Supplementary References**

1. Pastore A, Jurinovic V, Kridel R, Hoster E, Staiger AM, Szczepanowski M, et al. Integration of gene mutations in risk prognostication for patients receiving first-line immunochemotherapy for follicular lymphoma: a retrospective analysis of a prospective clinical trial and validation in a population-based registry. Lancet Oncol. 2015;16(9):1111-22.

2. Mootha VK, Lindgren CM, Eriksson K-F, Subramanian A, Sihag S, Lehar J, et al. PGC-1alph-responsive genes involved in oxidative phosphorylation are coordinately downregulated in human diabetes. Nature genetics. 2003;34:267.

3. Subramanian A, Tamayo P, Mootha VK, Mukherjee S, Ebert BL, Gillette MA, et al. Gene set enrichment analysis: A knowledge-based approach for interpreting genome-wide expression profiles. Proceedings of the National Academy of Sciences. 2005;102(43):15545-50.

4. Lu X, Nechushtan H, Ding F, Rosado MF, Singal R, Alizadeh AA, et al. Distinct IL-4-induced gene expression, proliferation, and intracellular signaling in germinal center B-cell-like and activated B-cell-like diffuse large-cell lymphomas. Blood. 2005;105(7):2924-32.

5. Schaefer CF, Anthony K, Krupa S, Buchoff J, Day M, Hannay T, et al. PID: the Pathway Interaction Database. Nucleic acids research. 2009;37(Database issue):D674-9.

6. Hadley W. ggplot2. New York, NY: Springer Science+Business Media, LLC; 2016. pages cm p.

7. Haebe S, Shree T, Sathe A, Day G, Czerwinski DK, Grimes SM, et al. Single-cell analysis can define distinct evolution of tumor sites in follicular lymphoma. Blood. 2021;137(21):2869-80.

8. Deliard S, Zhao J, Xia Q, Grant SF. Generation of high quality chromatin immunoprecipitation DNA template for high-throughput sequencing (ChIP-seq). Journal of visualized experiments : JoVE. 2013(74).

9. Cartharius K, Frech K, Grote K, Klocke B, Haltmeier M, Klingenhoff A, et al. MatInspector and beyond: promoter analysis based on transcription factor binding sites. Bioinformatics (Oxford, England). 2005;21(13):2933-42.

# **Supplementary Figure Legends**

**Suppl. Fig. 1:** Variant allele frequencies of selected recurrent mutations from three primary human FL harboring the *STAT6*^D419N^ polymorphism-like variant.

**Suppl. Fig. 2**: Visualizations of the sequencing reads spanning the *STAT6* mutational hotspot at D419 from two *STAT6*^MUT^ FL. Sequencing results of patient ID#1738 (top, GLSG cohort) and ID#RG071 (bottom, BCCA cohort), each with two distinct STAT6 mutations. Displayed is the BAM sequencing file by IGV Browser (Broad Institute).

**Suppl. Fig. 3: A)** *FCER2* mRNA levels (by qPCR) in OCI-Ly1 and OCI-Ly8 cells expressing either STAT6^WT^, STAT6^D419N^, STAT6^N421K^, STAT6^D519V^ or EV control after IL-4 stimulation (10 ng/mL, 24 hours; 2^-dCt^ values relative to STAT6^WT^, N = 3, mean ± SD). **B)** CD23 cell surface expression (by FACS) on OCI-Ly1 and OCI-Ly8 cells expressing either STAT6^WT^, STAT6^D419N^, STAT6^N421K^, STAT6^D519V^ or EV after IL-4 stimulation (10 ng/mL, 24 hours; geometric mean, N = 3, mean ± SD). **C)** Soluble CD23 (sCD23) levels (by ELISA) in cell culture supernatants of OCI-Ly1 and OCI-Ly8 cells expressing either STAT6^WT^, STAT6^D419N^, STAT6^N421K^, STAT6^D519V^ or EV after IL-4 stimulation (10 ng/mL, 72 hours by ELISA (N = 3, mean ± SD). **D)** *FCER2* mRNA levels and **E)** CD23 cell surface expression analogous to A) and B) after IL-4 pulse, i.e. IL-4 was added to cells for 20 minutes followed by washing and an additional 8 hour incubation without IL-4.

“-“ indicates no IL-4 stimulation, “+” indicates IL-4 stimulation (10 ng/mL for 20 minutes), and “P” indicates IL-4 pulse stimulation (IL-4 10 ng/mL for 20 minutes, then wash & withdrawal of IL-4 and incubation for another 8 hours in fresh media without IL-4).

**Suppl. Fig. 4: A)** Immunoblots of subcellular fractions from OCI-Ly1 and OCI-Ly8 cell lines expressing STAT6^WT^ and STAT6^D419N^ (analogous to **Fig. 2E)**. Lamin B1: nuclear loading control; α-Tubulin: cytoplasmic loading control. Legend: “-“, without IL-4; “+”, 20 min IL-4; “P”, IL-4 pulse (20 minutes IL-4, removal of IL-4, further incubation for 8 hours). **B)** Expression of 3xFlag-tagged STAT6 in stably transduced OCI-Ly1 and OCI-Ly8 cells. **C)** Immunoblots showing pSTAT6 and STAT6 levels in whole cell lysates from OCI-Ly1 STAT6^WT^ and STAT6^MUT^ cells.

“-“, without IL-4; “+”, 20 minutes IL-4; “P”, IL-4 pulse (20 minutes IL-4, removal of IL-4, further incubation for 8 hours). α-Tubulin was used as loading control.

**Suppl. Fig. 5: A)** Work flow of the whole transcriptome sequencing experiment. **B)** Principle component analysis of 54 samples from the RNA-seq experiment. Clustering according to *STAT6* mutational status and the different time points. **C)** Heatmaps of the top differentially expressed genes (log_2_FC change ±0.75, p-value < 0.0001) for the 2, 4 and 8 hour timepoints.

**Suppl. Fig. 6: Proximity ligation assay (PLA) of STAT6 and PARP14. A)** Representative images of OCI-Ly8 cells expressing STAT6^D419G^ with or without IL-4 stimulation; TexasRed channel used to detect red amplification signal. **B)** Box plots display the percentage of TexasRed positive cells per total cell number for each analyzed microscopic view field (n = 5)

**Suppl. Fig. 7: A)** UMAP representation of scRNA-seq data from 8 patients with FL. Cells are colored by cell type assignment (tumor cells in red). Numbers indicate tumor cells of individual patients. **B)** Dot plot showing expression (dot color) of canonical marker genes for the identified cell types. Dot size reflects the proportion of cells expressing the selected gene.

**Suppl. Fig. 8:** PARP14 promoter luciferase assay (pGL3) with co-transfected *STAT6*^WT^ or *STAT6*^D419G^ or *STAT6*^D419N^ in HeLa cells in the presence of IL-4 (24 hours after transfection, 10 ng/mL for 6 hours) with knock-down of PARP14 by shRNA (sh4 and sh5) or a non-targeting (scrambled) control (scr), respectively. Shown are fold changes (FC) of luciferase activity (relative light unit, RLU) normalized to 1 ng STAT6^WT^ (N = 3, mean ± SD). Immunoblot of respective cells as indicated below.
